# Epigenetic regulation of RhoB loss of expression in lung cancer

**DOI:** 10.1186/1471-2407-7-220

**Published:** 2007-11-30

**Authors:** Julien Mazières, Daniel Tovar, Biao He, Jacobo Nieto-Acosta, Claire Marty-Detraves, Carine Clanet, Anne Pradines, David Jablons, Gilles Favre

**Affiliations:** 1 INSERM U563 – Département Innovation Thérapeutique et Oncologie Moléculaire, F-31052, Toulouse, France; 2 Université Paul Sabatier, FB1062 Toulouse, France; 3 Institut Claudius Regaud, Toulouse, France; 4 CHU Toulouse, Hôpital Larrey, Service de pneumologie, 31059 France; 5 Thoracic Oncology Laboratory, Department of Surgery, Comprehensive Cancer Center, University of California, San Francisco, USA

## Abstract

**Background:**

RhoB is down-regulated in most lung cancer cell lines and tumor tissues when compared with their normal counterparts. The mechanism of this loss of expression is not yet deciphered.

**Methods:**

Since no mutation has been reported in the RhoB sequence, we investigated the epigenetic regulation of RhoB expression by analyzing the effect of HDAC inhibitors and methyltransferase inhibitors, by direct sequencing after bisulfite treatment and by methylation specific PCR.

**Results:**

We first showed that histone deacetylase (HDAC) inhibitors induce a significant RhoB re-expression in lung cancer cell lines whereas only a slight effect was observed with methyl transferase inhibitors. As promoter methylation is the most common epigenetic process in lung cancer, we performed methylation specific PCR and sequence analysis after bisulfite treatment and demonstrated that RhoB was methylated neither in lung cancer cell lines nor in tumor tissues. We also showed that a variable number of tandem repeats sequences in the 5' region of the RhoB gene was involved in HDAC response.

**Conclusion:**

We thus propose that RhoB regulation of expression occurs mainly by histone deacetylation rather than by promoter hypermethylation and that this process can be modulated by specific 5' sequences within the promoter.

## Background

Identification and characterization of genetic and epigenetic changes that drive lung cancer development and progression is of high interest for a better understanding of lung carcinogenesis. RhoB has been recently identified as a gene widely involved in lung carcinogenesis [1-3].

The small GTP binding protein RhoB belongs to the Rho subgroup (RhoA, B, and C) of the Rho protein family, which regulates diverse cellular processes including cytoskeletal organization, gene transcription, cell cycle progression, and cytokinesis [4,5]. Although RhoA and RhoB share 86% amino acid sequence identity, RhoB displays several distinct properties such as subcellular localization in endosomes and pre-lysosomal compartment [6], rapid turnover at a mRNA and protein level [7], post translational modification by either farnesylation or geranylgeranylation [8], and early upregulation by stress or growth factors [9,10]. Lastly, while most Rho proteins have been shown to have positive role in proliferation and malignant transformation processes, RhoB rather appears to act as a negative regulator [11,12].

It has been shown that ectopic expression of RhoB in human tumor cells led to an inhibition of tumor growth in nude mice [13] and that inactivation of RhoB in knock-out mice increased the frequency of tumors [14]. We recently showed that RhoB loss of expression occurred frequently in lung carcinogenesis [1]. We showed in two independent immunohistochemical studies that RhoB protein was expressed in normal lung and decreased dramatically through lung cancer progression. Interestingly, RhoB expression was lost in 96% of invasive tumors and reduced by 86% in poorly differentiated tumors compared with the non neoplastic epithelium. We also showed that ectopic expression of RhoB in lung cancer cell line A549 suppressed cell proliferation, anchorage-independent growth, and xenograft tumor growth in nude mice [1]. Loss of expression of RhoB has been reported in other solid tumors such as Head and Neck carcinomas [15] and brain tumors [16].

The mechanism by which RhoB expression decreases in lung carcinoma is not yet elucidated. The first hypothesis to be investigated is that RhoB loss of expression is due to genetic alterations such as mutation or deletion. In a previous study, Adnane *et al*. did not find any RhoB gene mutation in head and neck carcinoma [15]. Fritz *et al*. also reported that RhoA, RhoB, and RhoC were not altered by mutation in breast tumors [17]. More recently, Sato *et al*. showed that loss of heterozygosity (LOH) in the RhoB locus was found in 25 of 62 tumor samples analyzed [3] but correlation between LOH and RhoB loss of expression was not analyzed. The second hypothesis is that RhoB expression is controlled by epigenetic events. Wang *et al*. demonstrated that RhoB expression is repressed by histone deacetylase 1 (HDAC1) in lung cancer cell lines [2]. We previously reported the presence of a Variable Number of Tandem Repeat (VNTR) sequence in the human RhoB 5' region that is known to be linked with the penetrance and the development of several cancers [18].

In order to address specifically the epigenetic regulation of RhoB expression, we analyzed RhoB level of expression and promoter activity after treatment with demethylating agents and histone deacetylase inhibitors. Next, we performed RhoB promoter sequencing after bisulfite treatment and analyzed the involvement of the VNTR region in epigenetic regulation.

## Methods

### Cell lines and tumor tissues

Human lung carcinoma cells, A549, H460 and H838, mesothelioma cell lines, MS1 and H290 and breast cancer cell lines MCF-7 and BT474 were purchased from ATCC and were maintained in RMPI 1640 medium supplemented with 10% fetal calf serum (growth medium) at 37°C in a humidified incubator containing 5% CO_2_. BEAS-2B, bronchial cells immortalized by SV40 T antigen (ATCC CRL-9609), were maintained in DMEM (Dulbecco's Medium Modified) supplemented with 5% fetal calf serum at 37°C in a humidified incubator containing 5% CO_2_.

Fresh lung cancer tissues and adjacent normal lung tissues from patients undergoing resection at UCSF surgery department for lung cancers were collected at the time of surgery and immediately snap-frozen in liquid nitrogen (Institutional Review Board approval H8714-15319-040). These tissue samples were kept at -170°C in a liquid nitrogen freezer before use.

### Treatment of cells with HDAC inhibitors and demethylating agents

5-Azacytidine (Sigma, St. Louis, MO) treatment was performed as described previously [19]. Briefly, cells were treated for 72 hours with either 1, 3 or 10 μM of 5-Azacytidine each day. Trichostatin A (TSA) was purchased from Sigma (St. Louis, MO). Cells were treated with 1 μM TSA for 20 hours before analysis.

2'deoxy 5' azacytidine (Sigma, St. Louis, MO) treatment was performed as described for 5-Azacytidine with a concentration of 10 μM.

TSA plus 5-Azacytidine combination experiments were done by treating cells with 5-Azacytidine from day 1 to day 3 and TSA was added for the last 20 hours (at the same concentration than previously described).

### Reverse transcription PCR

Total RNA from lung cancer cell lines, fresh lung cancer, and paired adjacent normal tissue were isolated using an extraction kit (RNeasy Mini kit; Qiagen, Valencia, CA). Reverse transcription-PCR was performed in GeneAmp PCR system 9700 using One-step reverse transcription-PCR kit from Life Technologies, Inc., according to the manufacturer's protocol. Primers for reverse transcription-PCR were obtained from Operon Technologies, Inc. (Alameda, CA). Primer sequences for the human RhoB cDNA were 5'-TCGTAAGCCCAATTAAGGGGT-3' (forward) and 5'-GCTCTCTCCCGGGTCTCTCCG-3' (reverse) [18].

### Sequencing and methylation specific PCR (MSP)

Genomic DNA of the cell lines and fresh tissue samples was extracted using DNA STAT-60 reagent (TEL-TEST, Inc., Friendswood, TX), according to the manufacturer protocol. Bisulfite modification of genomic DNA was carried out by using a methylation kit (EZ DNA methylation kit; Zymo Research, Orange, CA). Bisulfite-treated genomic DNA was amplified using two pairs of primers: 5'-ATTTAAGTTGGGGGTTGGGAAGGG-3' (forward) and 5'-CAAAACAACAACTCCAACCAAAC-3' (reverse), designed to amplify nucleotides -1230 to -428 of the *RhoB *promoter region; and 5'-GAGGGGTAATTTTGAATGGGAGT-3' (forward) and 5'-CATAAAAACCRAACCCRAACAACA-3' (reverse), to amplify nucleotides -819 to +3 (the start codon ATG of *RhoB *is defined as +1).

MSP analysis was done on the same cell lines and tissues by using specific unmethylated primers: 5'-TTATTGTTTGAGTTTGTTGTTTGAGTTTGT6-3' (forward) and 5'-AACTACCACAACAAAAACAATAAAAACACA-3' (reverse) and specific methylated primers: 5'-CGTTCGAGTTTGTTGTTCGAGTTCGC-3' (forward) and 5'-CCGCGACGAAAACGATAAAAACGCG-3' (reverse).

The PCR products were extracted from the agarose gel using an extraction kit (QIAquick Gel Extraction kit; Qiagen) and were subsequently sequenced at the DNA-sequencing Core Facility of the University of California, San Francisco Cancer Center.

### Western-blot

Cells were washed in cold PBS and harvested in lysis buffer (Hepes 50 mM, pH 7.5, Triton X100 1%, Glycerol 10%, NaCl 10 mM, MgCl_2 _5 mM, NaF 25 mM, EGTA 25 mM, protease inhibitor cocktail (Sigma), sodium orthovadanate 2 mM, paranitrophenylphosphate (6.4 mg/ml). Cellular protein was quantitated by Bradford assay (Biorad), and 10 to 40 μg of the cleared lysates were separated on a 12.5% SDS-PAGE, and electro-transferred onto PVDF membranes (Amersham Pharmacia Biotech). PVDF membranes were incubated with polyclonal antibodies against RhoB (119, Santa Cruz Biotechnology). Detection was performed using peroxydase-conjugated secondary antibodies (Biorad) and chemiluminescence detection kit (ECL, Amersham Pharmacia Biotechnology).

### Reporter gene experiment

A549 and BEAS-2B cells were stably transfected with a firefly luciferase reporter gene controlled by the RhoB promoter. Cells were treated with 5-Azacytidine for 3 days or TSA for 1 day or not treated. Cells were then harvested in Passive Lysis Buffer (Promega) then firefly luciferase activities were analyzed using Luciferase^® ^Reporter Assay System (Promega) in a plate reading luminometer (Berthold). Proteins were extracted (Biorad) and the ratio of luciferase activity/protein quantity was calculated.

### Statistical analysis

The chi-square test was used to assess differences in reporter gene experiments (p < 0.05 was considered significant).

## Results

### RhoB loss of expression in lung cancer is reversed by histone acetylation inhibitors

**Figure 1:**
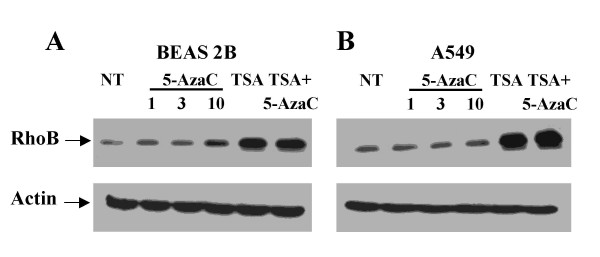
Western Blot analysis of RhoB expression in Beas-2B and A459 cell lines. Cells were either not treated (NT), treated with 5-Azacytidine (5-AzaC) at 1, 3 or 10 μM, with Trichostatin (TSA) at 1 μM or with combination of TSA (1 μM) and 5-AzaC (3 μM). Actin was used as a control. Experiments were performed 4 times with similar results.

We previously demonstrated RhoB loss of expression through lung cancer progression [1]. We reported that RhoB was expressed in normal and preinvasive tumors whereas its expression was weak and, most of the time, even lost in lung cancer invasive carcinoma [1]. No mutation has been found in Rho genes [15,17]. As many tumor suppressor genes are silenced by epigenetic modification in lung cancer [20], we hypothesized that RhoB expression might be repressed by either promoter methylation or histone deacetylation. We thus analyzed RhoB expression after treatment with a demethylating agent (5-Azacytidine) or a histone deacetylase inhibitor (Trichostatin A, TSA). We performed experiments in immortalized bronchial cells (BEAS-2B) and in lung cancer cell lines (A549). We first analyzed RhoB expression by Western-blot and showed in A549 lung cancer cell line that treatment by 5-Azacytidine did not significantly increase RhoB expression. At the opposite, we demonstrated that RhoB silencing in lung cancer cells was reversed by histone deacetylating agents. TSA and combination of TSA and 5-Azacytidine dramatically increased RhoB expression. The same tendency was observed in BEAS-2B but with less differential effect (Figure 1A &1B). As some authors propose that 2'-deoxy-5-azacytidine might be a more specific demethylating agent, we performed the same experiments with 2'-deoxy-5-azacytidine instead of 5-Azacytidine with the same results (data not shown). We thus demonstrated that demethylating agents cannot reverse RhoB loss of expression.

**Figure 2:**
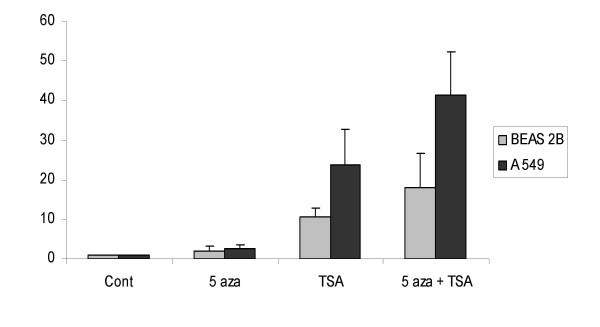
A549 and BEAS-2B cells were stably transfected with a firefly luciferase reporter gene controlled by the RhoB promoter. Cells were treated with 5 AzaC (10 μM) for 3 days, TSA (1 μM) for 1 day, combination of both or not treated (cont.). Luciferase activity was normalized with protein quantity.

We next analyzed luciferase activity in BEAS-2B and A549 stably transfected by a RhoB promoter construct. We first showed that 5-Azacytidine, TSA and combination of both were able to induce luciferase activity under the control of RhoB promoter by a factor of respectively 2, 10.6 and 17.9 in BEAS-2B and of 2.4, 23.8 and 41.5 in A549. There is an important differential effect between immortalized and lung tumor cell line. Moreover, it appeared that TSA was more potent than 5-Azacytidine to induce activity of RhoB promoter. Lastly, we demonstrated that 5-Azacytidine and TSA were synergistic in A549 cells (Fig 2).

### Analysis of RhoB promoter hypermethylation

**Figure 3:**
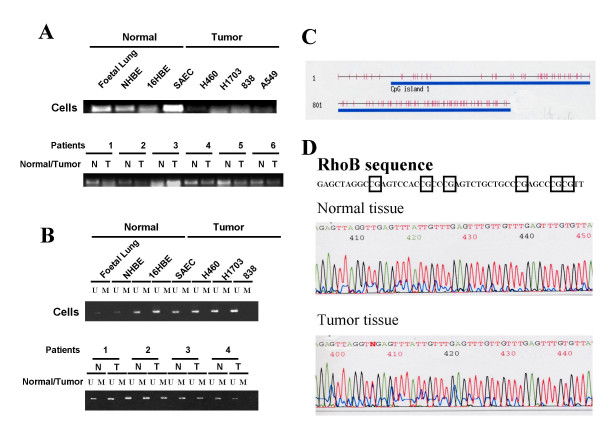
RhoB expression was analyzed in various normal and cancer cell lines by RT-PCR. Expression was also analyzed in normal (N) and tumor (T) tissues obtained from patient operated from lung cancer. B. MSP analysis was performed on DNA extracted from the same cell lines and from normal and tumor tissues. U and M designed PCR product obtained with Unmethylated or Methylated specific primers. C. Scheme of the 5' *RhoB *promoter region. The 5' region of *RhoB *was identified previously [18]. We used a CpG island searcher [29] that screens for CpG islands that meet the following criteria: CG percentage > 55%; observed CpG/expected CpG > 0.65; length > 500 bp. This rich-CpG islands region is *underlined*, and all CpG are represented by *vertical bars*. D. An example of RhoB sequence after bisulfite sequencing is shown with CpG island in square. Comparison between normal and tumor tissue is shown.

Epigenetic modification is a frequent mechanism of inactivation of tumor suppressor genes in lung cancer including promoter hypermethylation and histone deacetylation [21]. To unravel the respective role of these two main mechanisms, we analyzed the sequence of RhoB promoter after bisulfite treatment. We performed bisulfite treatment in various normal and tumor cell lines and in normal and tumor lung tissues. We first confirmed by Western Blot (not shown) and RT-PCR (Fig 3) that RhoB expression was lost or decreased in most lung cancer cells and in lung tumors when compared with normal cell lines or normal tissues. We first analyzed RhoB promoter [18] and isolated many CpG islands within its sequence. An *in silico *approach indicated the existence of two CpG-rich methylation-sensitive regions spanning from -415 to +430 and -814 to -507. We performed Methylation Specific PCR (MSP) by using primers designed to amplify specifically methylated or unmethylated region within the promoter region. A product of amplification was only obtained with unmethylated specific primers suggesting that the promoter was not methylated (Fig 3).

To check more carefully the status of each CpG in the 5' region, we designed specific primers to amplify CpG rich regions. Sequences analysis showed a few percentage of methylated CpG and, interestingly, no significant difference was observed either between normal cells and tumor cells or between normal and autologous tumor tissues (Table 1 &2, Fig 3). This data confirms that promoter hypermethylation is not the main mechanism of RhoB inactivation in lung cancer.

**Table 1:** Percentage of methylated CpG in various normal and tumor cell lines. Sequence analysis was performed after bisulfite treatment.

**Cells**	**Tumor type**	**meth CpG**
CCL-75	Fibroblast	5%
**NHBE**	Normal	6.3%
**SAEC**	Normal	4.5%
**A460**	Large cell	7.9%
**H838**	Adenocarcinoma	8.2%
**H1703**	Squamous cell	6.6%
**A549**	Adenocarcinoma	5.1%
**MS1**	Mesothelioma	2.2%
**H290**	Mesothelioma	4%
**MCF-7**	Breast cancer	1.6%
**BT474**	Breast cancer	2%

**Table 2:** Percentage of methylated CpG in tumor vs. normal tissue. Sequence analysis was performed after bisulfite treatment.

**Patients**	**Tissue**	**meth CpG**
**1**	Normal	2.5%
	Tumor	4%
**2**	Normal	2%
	Tumor	2.2%
**3**	Normal	3%
	Tumor	4.2%
**4**	Normal	5%
	Tumor	4.3%
**5**	Normal	1.5%
	Tumor	1.8%
**6**	Normal	4.9%
	Tumor	4.8%

### Involvement of RhoB VNTR in epigenetic regulation

**Figure 4:**
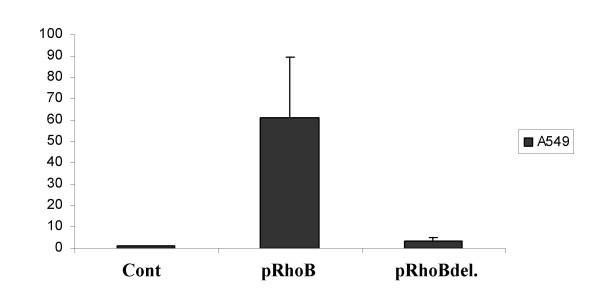
The same luciferase assay is performed as in figure 2. Cells were treated with 1 μM TSA for 72 hours. pRhoB designs wild type promoter and pRhoBdel. designed VNTR truncated promoter (details about the construct in Tovar 2003 [18]).

We previously characterized the 5'-flanking region of RhoB and found that it contains a Variable Number of Tandem Repeats (VNTR) sequence that affects transcriptional activity [18]. We thus analyzed if this VNTR sequence was involved in RhoB re-expression by HDAC inhibitors. We used deletion mutants of the 5' region containing the VNTR sequences as described in our previous work [18] and showed that TSA treatment in A549 cells transfected with RhoB and a wild type promoter induces an average of 60-fold RhoB re-expression whereas, when transfected with 5' deleted promoter, re-expression was weak (around 3.5-fold) (Fig 4). This suggests that the VNTR containing region is involved in RhoB re-expression by HDAC inhibitors.

## Discussion

Downregulation of RhoB has been shown in lung cancer cell lines [2] and lung cancer tissues [1,3] leading us to investigate the mechanisms leading to its loss of expression. As many genes are mutated in lung carcinomas (K-Ras, EGFR for example), mutational analysis of RhoB sequence has been performed by several teams in various tumors [3,15,17] and were all negative. Sato *et al*. found an allelic loss of RhoB in 40% of the analyzed cases. Nevertheless, it is not known if deletions were correlated with RhoB level of expression and what were the mechanisms of loss of expression for the other 60% of patients. It should be noticed that no deletion has been found in the RhoB gene in 12 head and neck carcinomas analyzed in another study [15].

Aberrant methylation of the promoter region of tumor suppressor genes and the resultant gene silencing play an important role in many cancers and especially in lung cancer initiation and progression [22]. Many suppressor genes such as p16, APC, Wif-1, RASSF1A [20,23,24] are regulated through promoter hypermethylation. We found that the RhoB promoter was rich in CpG dimers suggesting that methylation might be a possible mechanism of regulation. Moreover, we observed that treatment of lung cancer cells with 5-Azacytidine induced a slight increase in RhoB expression. Nevertheless, neither MSP analysis, nor promoter sequencing after bisulfite treatment allowed us to find CpG aberrant methylation. The same findings were observed in lung cancer cell lines and matched normal and tumor tissues. The slight effect observed with 5-Azacytidine might be due to regulation of other genes upstream of RhoB. RhoB loss of expression thus appears to be independent of promoter hypermethylation. These results are consistent with data showing that gene hypermethylation is rather an early event (as demonstrated for p16) [25] whereas RhoB loss of expression has been shown to occur lately in cancer progression [1,15]. Nevertheless, gene hypermethylation can also occur later through tumorigenesis.

Another major epigenetic process involved in the control of gene expression is histone deacetylation. Using a differential DNA microarray analysis, Wang *et al*. showed that RhoB gene was upregulated in response to Trapoxin (an HDAC inhibitor) treatment. More precisely, they demonstrated that HDAC1 repressed RhoB promoter [2]. In their recent study, Sato *et al*. also found that HDAC inhibitor treatment induced RhoB re-expression in 3 lung cancer cell lines [3]. Moreover, HDAC1 expression in lung cancer tissues has been shown to be correlated with cancer progression [26]. We can speculate that changes in HDAC activity through lung cancer progression might control expression of various genes involved in lung carcinogenesis. We found that RhoB expression was lost lately in lung carcinoma and was correlated with tumor stage [1]. This might be due to changes in HDAC expression in lung tissues. A correlation analysis between HDAC and RhoB expression in lung cancer is currently conducted in our laboratory. In summary, we found that RhoB gene expression is controlled by histone deacetylation rather than by methylation and that inhibition of both mechanisms was synergistic. These two processes are linked and synergy between demethylation and histone deacetylase inhibition in the re-expression of genes silenced in lung cancer has already been reported [21].

Regulation of RhoB expression in lung cancer appears to be complex and controlled by more than one mechanism. According to our work and to the literature, the main mechanisms are epigenetic regulation through histone deacetylation and genetic deletion. At the opposite, gene mutation and promoter hypermethylation have not been reported. Epigenetic events might coexist with genetic alterations. For example, in lung cancer, p16 is known to be regulated by gene deletion, missense mutation or promoter methylation [23]. The exact correlation between RhoB loss of expression and the genetic or epigenetic events has not been precisely studied yet and the respective roles of these various mechanisms remain unclear. We can hypothesize that regulation differs according to tumor stage or cancer type as proposed for other genes [20].

Another level of regulation relies upon the presence of specific sequences within the 5' region of the RhoB promoter that appear to be involved in the HDAC response. We isolated VNTR sequences located from -1124 to -821 that influence the transcriptional activity of the promoter [18]. Here, we show that RhoB expression induced by HDAC inhibitors is no more observed if the 5' region containing the VNTR sequences is deleted. The influence of a polymorphic VNTR sequence in human HRas on the risk or the penetrance of several cancers has been reported [27,28]. Wang *et al*. also reported that the induction of RhoB by HDAC inhibition was mediated by an inverted CCAAT box located in the RhoB promoter at the -451 position [2].

## Conclusion

We thus propose that RhoB regulation of expression occurs mainly by histone deacetylation rather than by promoter hypermethylation and that this process can be modulated by specific 5' sequences within the promoter. We can hypothesize that reversing RhoB loss of expression with HDAC inhibitors might be of therapeutic interest.

## Competing interests

The author(s) declare that they have no competing interests.

## Authors' contributions

JM and DT designed and carried out the cell lines studies and drafted the manuscript. They contributed equally to the manuscript. BH and DJ participated in the promoter methylation analysis at the Thoracic Oncology Laboratory at UCSF. They also revised the manuscript JNA, CC and CMD performed some of the reporter gene experiment and expression experiments. AP and GF conceived and coordinated the study and revised the manuscript. All authors read and approved the final manuscript.

## Pre-publication history

The pre-publication history for this paper can be accessed here:


